# Safety, Immunogenicity, and Efficacy of a Recombinant Vesicular Stomatitis Virus Vectored Vaccine Against Severe Fever with Thrombocytopenia Syndrome Virus and Heartland Bandavirus

**DOI:** 10.3390/vaccines12121403

**Published:** 2024-12-12

**Authors:** Philip Hicks, Tomaz B. Manzoni, Jonna B. Westover, Raegan J. Petch, Brianne Roper, Brian B. Gowen, Paul Bates

**Affiliations:** 1 Department of Microbiology, Perelman School of Medicine, University of Pennsylvania, Philadelphia, PA 19104, USA; hicksp@udel.edu (P.H.); manzonitb@gmail.com (T.B.M.); rpetch@vet.upenn.edu (R.J.P.); brianne.roper@pennmedicine.upenn.edu (B.R.); 2 Department of Animal, Dairy and Veterinary Sciences, Utah State University, Logan, UT 84322, USA; jonna.westover@usu.edu (J.B.W.); brian.gowen@usu.edu (B.B.G.); 3 Institute for Antiviral Research, Utah State University, Logan, UT 84322, USA

**Keywords:** bandavirus, severe fever with thrombocytopenia syndrome virus, heartland virus, heartland bandavirus, Dabie bandavirus, vaccine, rVSV, vesicular stomatitis virus, immunity, neutralizing antibodies, tick-borne virus

## Abstract

Background: Severe fever with thrombocytopenia syndrome virus (SFTSV) is a recently emerged tickborne virus in east Asia with over 18,000 confirmed cases. With a high case fatality ratio, SFTSV has been designated a high priority pathogen by the WHO and the NIAID. Despite this, there are currently no approved therapies or vaccines to treat or prevent SFTS. Vesicular stomatitis virus (VSV) represents an FDA-approved vaccine platform that has been considered for numerous viruses due to its low sero-prevalence in humans, ease in genetic manipulation, and promiscuity in incorporating foreign glycoproteins into its virions. Methods: In this study, we developed a recombinant VSV (rVSV) expressing the SFTSV glycoproteins Gn/Gc (rVSV-SFTSV) and assessed its safety, immunogenicity, and efficacy in C57BL/6, *Ifnar^−/−^*, and AG129 mice. Results: We demonstrate that rVSV-SFTSV is safe when given to immunocompromised animals and is not neuropathogenic when injected intracranially into young immunocompetent mice. Immunization of wild type (C57BL/6) and *Ifnar^−/−^* mice with rVSV-SFTSV resulted in high levels of neutralizing antibodies and protection in a lethal SFTSV challenge model. Additionally, passive transfer of sera from immunized *Ifnar^−/−^* mice into naïve animals was protective when given pre- or post-exposure. Finally, we demonstrate that immunization with rVSV-SFTSV cross protects AG129 mice against challenge with the closely related Heartland bandavirus despite negligible neutralizing titers to the virus. Conclusions: Taken together, these data suggest that rVSV-SFTSV is a promising vaccine candidate for SFTSV and Heartland bandavirus with a favorable safety profile.

## 1. Introduction

Severe fever with thrombocytopenia syndrome (SFTS) is an emerging tickborne disease caused by the SFTS virus (SFTSV, recently renamed *Dabie bandavirus*, formerly known as *Huaiyangshan Banyangvirus*), which was first identified in 2011 in China [1]. As of 2021, more than 18,000 cases had been confirmed [2], and SFTSV has now been detected in patients throughout Japan, South Korea, Vietnam, Taiwan, Pakistan, Myanmar, and Thailand [3,4,5,6,7,8,9,10,11,12]. Infected patients present with fever, thrombocytopenia, and leukopenia, which can progress to hemorrhagic tendency and multi-organ failure resulting in death [1,13,14]. The overall case fatality rate is approximately 8% [2], but small outbreaks have had fatality rates as high as 47% [8], and annual infections have increased in number since identification of the virus [2,15,16,17]. In 2009, a novel bunyavirus named Heartland bandavirus (HRTV) was discovered in Missouri, exhibiting a similar disease progression and transmission cycle to SFTSV [18]. HRTV was later shown to be closely related to SFTSV [18]. According to the Centers for Disease Control and Prevention (CDC), more than 60 cases of HRTV infections have since been detected throughout the eastern and midwestern United States with an estimated 5–10% fatality rate [19]. However, HRTV is not a notifiable disease in the United States, and therefore case numbers are likely significantly underreported.

SFTSV and HRTV are both bandaviruses in the class *Bunyaviracetes*, order *Hareavirales*, and family *Phenuiviridae* [20]. These viruses have a trisegmented, single-stranded RNA genome encoding four proteins. The S segment is ambisense and encodes the nucleoprotein (N) in the negative sense and a nonstructural protein (NSs) in the positive sense [1]. The L and M segments are negative sense and encode the RNA-dependent RNA polymerase (RdRp) and envelope glycoproteins, respectively. The glycoprotein is translated as a polyprotein, which is proteolytically cleaved into two subunits, Gn and Gc [1,21]. Gn facilitates viral attachment via receptor recognition, and entry is mediated by the fusion peptide within Gc [22,23,24,25,26]. While studies have failed to isolate a definitive receptor for SFTSV Gn/Gc, some molecules have been identified as important binding or entry factors, including DC-SIGN, UGCG, non-muscle myosin heavy chain IIA, and CCR2 [22,23,25,27,28]. Entry is less well understood for HRTV, but C-type lectins, DC-SIGN, DC-SIGNR, LSECtin, and UGCG have been shown to be involved [29,30]. Antibodies against both Gn and Gc have been shown to inhibit viral entry into cells, but Gn is the primary target of neutralizing antibodies [23,31,32,33,34,35].

Tick exposure is the primary mode of both SFTSV and HRTV infection. The primary vector for SFTSV is the tick *Haemaphysalis longicornis,* which is found throughout eastern Asia [36,37]. However, SFTSV has been found in other tick genera as well, including *Ixodes* and *Amblyomma*, suggesting that numerous tick species might transmit this pathogen [38,39]. In recent years, the geographic distribution of *H. longicornis* has expanded and now includes Australia, New Zealand, and the United States, presenting further opportunities for SFTSV to spread [40,41,42,43]. Although rare, other transmission routes have been shown to be possible for SFTSV. Ferret studies have shown that SFTSV can be transmitted in the absence of ticks between co-housed ferrets or ferrets co-housed with a separator [44]. The detection of SFTSV in ferret saliva, feces, and urine suggests that these fluids are a likely route of SFTSV transmission in the absence of ticks [44]. Indeed, reports indicate that SFTSV can spread between humans in nosocomial settings through contact with patient blood, respiratory secretions, or other bodily fluids [45,46,47,48,49]. Additional case reports showed likely zoonotic transmission to humans via infected cats [50,51,52,53]. The primary vector for HRTV is the tick *Amblyomma americanum*, although there is evidence that the newly introduced *H. longicornis* could also serve as a vector for HRTV in the United States [54,55]. Due to the high case fatality rate of SFTSV, the expanding host range of its vector, and the potential for zoonotic and human-to-human transmission, SFTSV has been categorized as a high priority pathogen for the development of vaccines and therapeutics by both the World Health Organization (WHO) and the National Institute of Allergy and Infectious Diseases (NIAID) [56,57]. Similarly, the emergence and high case fatality rate of HRTV has drawn concern from the NIAID, and they have identified it as a Category C priority pathogen [57].

Despite prioritization by the WHO and NIAID, currently no approved therapeutics or vaccines exist for use against SFTSV or HRTV. Several groups have designed and tested SFTSV vaccines, primarily using *Ifnar^−/−^* mice. Tested vaccine platforms include DNA, mRNA, protein subunit, whole inactivated virus, live attenuated virus, and virus-vectored vaccines [58,59,60,61,62,63,64,65,66,67,68,69,70]. These vaccines vary in their effectiveness and come with drawbacks. The majority of SFTSV vaccines that have been developed utilize Gn and Gc glycoproteins as they are known to be the primary target of neutralizing antibodies [31,32,33,34,35], and anti-Gn neutralizing antibodies are a correlate of protection in infected patients [71]. Additionally, vaccination with Gn or Gc induces cellular immunity. In contrast, the SFTSV nucleoprotein, nonstructural protein, and RNA dependent RNA polymerase do not elicit neutralizing antibodies but instead elicit protective T cell responses upon vaccination [58]. HRTV vaccine development lags behind that of SFTSV, but early work has explored production of virus-like particles expressing HRTV proteins as a first step in the development of vaccines [72]. Additionally, SFTSV mRNA vaccines have been shown to cross protect against lethal HRTV challenge [62]. Here, we focus on developing and characterizing a recombinant vesicular stomatitis virus (rVSV) vaccine that can provide protection against lethal challenge with both SFTSV and HRTV.

The livestock pathogen vesicular stomatitis virus (VSV) is generally non-pathogenic to humans and of low sero-prevalence [73,74]. Additionally, VSV is a powerful vaccine platform with genetically tractable models and a promiscuity to incorporate foreign glycoproteins in the virion [75]. An often-cited detriment of rVSV vaccines is the propensity for VSV to be neurotropic. It is, however, known that neuropathogenicity is conferred by the tropism of the viral glycoprotein [76,77,78,79,80], which is replaced entirely with foreign glycoproteins in current vaccine platforms [75,81]. Currently, the rVSV vaccine platform is approved for use against Ebola virus (EBOV) and has been successfully distributed to more than 350,000 people in Africa during recent EBOV outbreaks [82,83,84]. Due to the proven nature of the rVSV platform and the cellular and humoral immunogenic potential of the SFTSV glycoproteins, we produced a rVSV-SFTSV virus containing the SFTSV Gn/Gc glycoprotein in place of the cognate VSV glycoprotein VSV-G.

It has been previously reported by another group that rVSV-SFTSV confers protective immunity to *Ifnar^−/−^* mice [67]. To go beyond what has been previously shown, we demonstrate that our rVSV-SFTSV is non-neurotropic and safe in immunocompromised animals. We also show that a single administration of vaccine virus is sufficient to induce protection against SFTSV challenge. Additionally, rVSV-SFTSV vaccination induces high levels of antibodies in wild-type animals, suggesting that it can effectively be used in immune competent animals. Both therapeutic and prophylactic passive transfer of sera from immunized animals leads to protection upon challenge of unvaccinated animals, suggesting that antibodies correlate with protection against SFTSV. Finally, we demonstrate that our rVSV-SFTSV vaccine is cross protective upon lethal HRTV challenge.

## 2. Materials and Methods

### 2.1. Cells, Viruses, and Mice

ATCC verified and mycoplasma free 293T and Vero E6 cells were maintained in DMEM (Corning, Tewksbury, MA, USA, #10-013-CV) containing 10% FBS (Corning, #35-010-CV), and 2 mM L-glutamine (Corning, #25-005-Cl). Cells were passaged every 2–3 days.

Recombinant viruses harboring an additional open reading frame encoding EGFP (referred to as VSV throughout this paper) in genomic position 5 or encoding heterologous viral glycoproteins in genomic position 4 (rVSV-SFTSV-HB29 and rVSV-EBOV) were launched and described previously [27,85]. rVSV-SFTSV and rVSV-EBOV also contain an additional open reading frame in position 5, encoding mCherry as previously described for rVSV-EBOV [85,86]. All recombinant viruses were grown in Vero E6 cells by infecting a confluent T-175 flask at a multiplicity of infection (MOI) of 0.3–0.5. Virus was collected at 48–72 h post-infection with the addition of Hepes buffer pH 7.4 to 25 mM. Media were clarified by centrifuging twice at 6000 times gravity for 5 min at 4 °C. Virus was then frozen at −80 °C until used for ultracentrifugation. Virus was concentrated by ultracentrifugation of virus-containing media through a 20% sucrose gradient at 26,000 rpm for 2 h at 4 °C using SW-32 tubes in a Beckman Coulter Optima XPN-80 ultracentrifuge (Beckman Coulter, Brea, CA, USA). After removal of the sucrose and media, pelleted virus was placed on ice with 500 µL Hepes buffered saline overnight. The next day virus pellets were resuspended and frozen at −80 °C. Viral titer was determined by plaque assays on Vero E6 cells with a 1.25% Avicel RC-591 NF (DuPont, Newark, DE, USA, #RC591-NFBA500) overlay and then stained with 1% crystal violet. Due to the size difference of plaques created by VSV and rVSV-SFTSV, plates were processed at 36 or 72 h post infection, respectively, unless otherwise stated.

SFTSV, strain HB29, was obtained from Dr. Robert Tesh (WRCEVA; World Reference Center for Emerging Viruses and Arboviruses at the University of Texas Medical Branch, Galveston, TX, USA). The virus stock (5.6 × 10^6^ plaque-forming units [PFU]/mL; 1 passage in Vero E6 cells) used was from a clarified cell culture lysate preparation. Virus stock was diluted in sterile minimal essential medium (MEM) and inoculated by subcutaneous injection of 0.2 mL containing approximately 10 PFU.

The mouse-adapted HRTV (MA-HRTV) strain employed was derived from the MO-4 strain obtained from Dr. Robert Tesh (WRCEVA) [87]. The MA-HRTV stock (4.7 × 10^8^ 50% cell culture infectious dose (CCID_50_/mL); 1 passage in Vero E6 cells, 5 passages in AG129 mice) used was prepared from clarified liver homogenate. The virus stock was diluted in sterile MEM and inoculated bilaterally in two intraperitoneal (IP) injections of 0.1 mL each for a total inoculation of 40 CCID_50_.

C57BL/6 mice were ordered from Jackson Labs (Bar Harbor, ME, USA). AG129 (IFN-α/β and γ receptor-deficient) and *Ifnar^−/−^* mice were obtained from breeding colonies at Utah State University. Four-week-old C57BL/6 mice were used for intracranial challenge experiments. Eight-week-old C57BL/6 mice or *Ifnar^−/−^* mice on the C57BL/6 background were used for all other experiments. All mouse experiments were conducted using equal numbers of male and female mice. All mice were given approximately 7 days to acclimate to their cages and vivarium prior to each experiment. Mice were weighed immediately prior to all vaccination and infection procedures. All mice were anesthetized using 1% isoflurane in air delivered by vaporizer (Northern Vaporisers, Skipton, UK) to the anesthesia chamber. Injection sites were first prepared by cleaning with a 70% ethanol pad. Intracranial injection experiments and some vaccination experiments without authentic SFTSV challenge were performed under animal biosafety level (ABSL) 2 conditions at the University of Pennsylvania. All other vaccination experiments that included authentic SFTSV challenged were performed in ABSL3 conditions at Utah State University.

All animals were treated ethically, complying with guidelines set by the USDA, the Utah State University Institutional Animal Care and Use Committee, and the University of Pennsylvania Laboratory Animal Resources guidelines.

### 2.2. Western Blot

Vero E6 cells were mock infected or infected with rVSV-SFTSV. At 24 h post infection, cells were lysed, Laemmli buffer was added, and samples were denatured at 95 °C for 5 min. Samples were run on a 4–15% Biorad gel (Bio-Rad Laboratories, Hercules, CA, USA, #5671084). Protein was transferred to a PVDF membrane (Millipore Sigma, Burlington, MA, USA, #IPVH00010) and stained with anti-SFTSV Gn (ProSci, Fort Collins, CO, USA, #6647) or Gc (ProSci, #6651) polyclonal antibodies followed with a secondary HRP conjugated antibody (GE Healthcare, Chicago, IL, USA, #NA934V). Between stainings, membrane was stripped for an hour at room temp using restore Western blot stripping buffer (Thermo Fisher Scientific, Waltham, MA, USA, #21059). Western blots were developed using SuperSignal West Pico PLUS Chemiluminescent Substrate (Thermo Fisher Scientific, #34580) and read on a GE Healthcare Amersham 600 imager (GE Healthcare). Band intensity was analyzed via FIJI (ImageJ, https://ij.imjoy.io, accessed on 2 December 2024).

### 2.3. rVSV-SFTSV Replication Kinetics Assay

Vero E6 cells were infected at a MOI of 0.01 with rVSV-SFTSV or VSV diluted in DMEM + 10% FBS for 2 h. The inoculum was then removed, and cells were gently washed three times with PBS. Cells were then covered in fresh complete growth medium and incubated at 37 °C. Every 24 h, 5% of the growth medium was removed and replaced with an equal volume of fresh growth medium. Samples were clarified by centrifugation, transferred to fresh tubes, and frozen at −80 °C until they were titrated by plaque assay as previously described [88].

### 2.4. Measurement of Plaque Area

Wells were imaged individually using a GelDoc XR+ with Image Lab Software version 5.1 (Bio-Rad Laboratories, Hercules, CA, USA) with Coomasie Blue settings. Images were analyzed using FIJI (ImageJ, available online: https://ij.imjoy.io, accessed on 14 March 2022). First, images were thresholded using a pixel intensity cutoff of 216. Thresholded images were then converted to binary masks. Regions of interest were automatically drawn around plaques using the “Analyze Particles” command. Regions of interest that contained two or more plaques were discarded and redrawn using the “polygon ROI” tool such that regions of interest only included a single plaque. The area of each region of interest was then measured using the “Measure” tool.

### 2.5. Immunization

Vaccines were diluted to the desired concentrations with sterile PBS immediately prior to vaccination by IP injection. All immunizations were conducted with a 200 µL inoculum. Favipiravir, the positive control for the rVSV-SFTSV vaccine efficacy study, was kindly provided by the Toyama Chemical Co., Ltd. (Toyama, Japan) and prepared in a meglumine solution for administration by IP injection.

### 2.6. Intracranial Infection and Neurologic Sign Scoring

To evaluate neuropathogenesis, 4-week-old C57BL/6 mice were injected intracranially into the right cerebral hemisphere using a 1 mL Hamilton syringe with Repeating Syringe Dispenser (Hamilton Company, Reno, NV, USA). Inocula contained 0, 10^1^, 10^2^, or 10^3^ PFU of rVSV-SFTSV or rVSV-EGFP and were diluted to a total injection volume of 10 μL with PBS. Mice were monitored during anesthesia recovery until they were ambulatory. Mice were weighed daily and were observed for neurologic signs. Neurologic signs were assigned a severity score ranging from 0–4. Mice scored “0” showed no signs of illness and were bright, alert, and responsive when handled. Mice scored “1” showed mild signs of illness without clear signs of neurologic illness, including body hunching, depressed activity, or mild grimace. Mice assigned a “1” had normal ambulation and responded normally to being handled. Mice assigned a “2” had clinical signs consistent with mild encephalitis, including hyperexcitability or altered gait that did not impair linear locomotion and used all four limbs. Mice assigned a “3” had more severe neurologic signs, which included paraparesis of one or two limbs, mild head tilt, and altered gait that did impair linear locomotion (such as spinning). Mice assigned a “4” had severe neurologic signs that were inconsistent with life, including complete pelvic limb paraplegia, ataxia, or tremors/seizures. Mice scored with a “4” were humanely euthanized with CO_2_.

### 2.7. Blood Collection

Mice were isoflurane anesthetized, and blood was collected through the submandibular route using Goldenrod lancets 5mm (Medipoint, Mineola, NY, USA). Blood was maintained on ice after collection. Serum was separated from blood by centrifugation at 8000 RPM for 30 min at 4 °C in an Eppendorf 5424R centrifuge (Eppendorf, Enfield, CT, USA). Serum was heat inactivated by incubating at 56 °C for 30 min. While running neutralization assays, serum was stored at 4 °C. For long term storage, serum was frozen at −80 °C.

### 2.8. Pseudovirus Neutralization Assay

Production of VSV pseudotype with SFTSV Gn/Gc: 293T cells plated 24 h previously at 2 × 10^7^ cells per T-175 flask were transfected using Lipofectamine 2000 (Invitrogen, Waltham, MA, USA, #11668-019) using the manufacturer’s protocol. Briefly, tubes, each containing 1.75 mL Opti-MEM (Gibco, Waltham, MA, USA, #31985-070), were produced. In one tube, 100 μL of Lipofectamine 2000 reagent was added and gently mixed. In the other, 45 μg of pCAG-SFTSV Gn/Gc expression plasmid was added. Tubes were allowed to sit for 5 min at room temperature. Lipofectamine and DNA containing tubes of Opti-MEM were combined and gently mixed after 20 min incubating at room temperature. The solution was added to a flask of 293T cells, and after 4 h cells were fed with fresh media. Thirty hours after transfection, the SFTSV Gn/Gc expressing cells were infected for 2–4 h with VSV-G pseudotyped VSVΔG-mNeon at an MOI of ~1–3. VSVΔG-mNeon was generated by deleting the cognate VSV-G and linking mNeon to the n-terminus of P, and virus was launched as previously described [27]. After infection, the cells were washed twice with FBS-free media to remove unbound virus. Media containing the VSVΔG-mNeon SFTSV Gn/Gc pseudotypes were harvested 30 h after infection and clarified by centrifugation twice at 6000× *g* then aliquoted and stored at −80 °C until used for antibody neutralization analysis.

Antibody neutralization assay using VSVΔG-mNeon SFTSV Gn/Gc: Vero E6 cells were seeded in 100 μL at 2 × 10^4^ cells/well in a 96-well collagen coated plate. The next day, 2-fold serially diluted serum samples were mixed with VSVΔG-mNeon SFTSV Gn/Gc pseudotype virus (100–200 focus forming units/well) and incubated for 1 h at 37 °C. Also included in this mixture, to neutralize any potential VSV-G carryover virus, was 1E9F9, a mouse anti-VSV Indiana G, at a concentration of 600 ng/mL. The antibody-virus mixture was then used to replace the media on Vero E6 cells. Then, 16 h post infection, the cells were washed and fixed with 4% paraformaldehyde before visualization on an S6 FluoroSpot Analyzer (CTL, Shaker Heights, OH, USA). Individual infected foci were enumerated, and the values compared to control wells without serum. The focus reduction neutralization titer 50% (FRNT_50_) was measured as the greatest serum dilution at which focus count was reduced by at least 50% relative to control cells that were infected with pseudotype virus in the absence of mouse serum. FRNT_50_ titers for each sample were measured in two to three technical replicates performed on separate days.

### 2.9. Virus Titer Determination

Virus titers were assayed using an infectious cell culture assay as previously described [89]. Briefly, a specific volume of tissue homogenate or serum was serially diluted and added to triplicate wells of Vero E6 (African green monkey kidney) cell monolayers in 96-well microtiter plates. The viral cytopathic effect (CPE) was determined 11 days after plating and the 50% endpoints calculated as described in [90]. The lower limits of detection were 1.67 log_10_ CCID_50_/mL serum and 2.43–3.14 log_10_ CCID_50_/g tissue. In samples presenting with virus below the limits of detection, a value representative of the limit of detection was assigned for statistical analysis.

### 2.10. Passive Transfer

The immune sera from mice vaccinated with rVSV-SFTSV (approximate FRNT_50_ of 453), non-immune sera, and recombinant vaccine rVSV-SFTSV (7.12 × 10^7^ PFU/mL) were diluted with sterile PBS so that the volume of each treatment was 100 µL. Sera was delivered by IP injection 2 days prior to or post challenge with SFTSV. Mice receiving the rVSV-SFTSV vaccine were immunized 7 days prior to challenge. Monitoring of mouse weight began at 7 days prior to challenge and continued 21 days post SFTSV challenge.

### 2.11. Statistical and Data Analysis

The Mantel–Cox log-rank test was used for analysis of Kaplan–Meier survival curves. A one-way analysis of variance (ANOVA) with Dunnett’s post test to correct for multiple comparisons was used to assess differences in virus titers. A one-way ANOVA with Tukey’s multiple comparisons post-hoc test was used to assess FRNT_50_ titers and maximum neurologic sign scores. Unpaired student’s t-test with unequal variance was used to assess differences in plaque area. All statistical evaluations were conducted using Prism 9 (GraphPad Software, La Jolla, CA, USA).

## 3. Results

### 3.1. rVSV-SFTSV Is Attenuated in Ifnar^−/−^ Mice and Exhibits a Favorable Safety Profile

**Figure 1:**
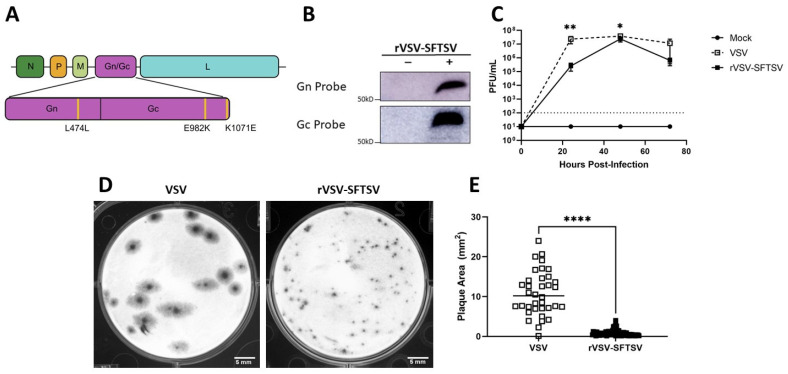
rVSV-SFTSV expresses SFTSV glycoproteins and is attenuated in vitro. (**A**) Schematic of mutations in SFTSV Gn/Gc that arose during passage. (**B**) Expression of SFTSV Gn and Gc by cells infected with rVSV-SFTSV. Gn band intensity: 94.86, Gc band intensity: 137.47. The uncropped, unedited blots are shown in Supplementary Materials. (**C**) Growth kinetics of rVSV-SFTSV and VSV in Vero E6 cells infected at a multiplicity of infection of 0.01. (Two-way ANOVA with Tukey’s multiple comparisons test; *, *p* < 0.0458; **, *p* < 0.0024). Images (**D**) and surface area (**E**) of plaques created by VSV and rVSV-SFTSV on Vero E6 cell monolayers 48 h post infection. (Unpaired t-test with unequal variance; ****, *p* < 0.0001).

We launched rVSV-SFTSV in HEK293T cells as previously described [12]. The recovered virus was expanded on Vero E6 cells for three passages to collect a vaccine stock. A sample of the virus was sequenced to assess mutations in the SFTSV glycoprotein that arose during launch and expansion. Three point mutations were identified in the gene encoding SFTSV Gn/Gc. These mutations included a synonymous mutation and two nonsynonymous mutations that caused the substitutions E982K and K1071E in Gc (Figure 1A). Expression of SFTSV Gn and Gc was confirmed by Western blot of cell lysates prepared from Vero E6 cells infected with rVSV-SFTSV (Figure 1B).

Before performing in vivo studies, we first determined if rVSV-SFTSV was attenuated in cell cultures relative to parental rVSV. To evaluate growth kinetics, Vero E6 cells were inoculated at an MOI of 0.01 with rVSV-SFTSV or rVSV. Cell supernatants were sampled every 24 h and infectious virus was titrated by plaque assay. At 24 h post infection, infectious titers of rVSV were nearly 90-fold higher than titers of rVSV-SFTSV (Figure 1C). Both viruses achieved similar maximum titers by 72 h post infection. rVSV-SFTSV caused a cytopathic effect in Vero E6 cells as evidenced by cell rounding and detachment, as well as the formation of plaques on Vero E6 cell monolayers by 48 h post infection (Figure 1D). However, the plaques created by rVSV-SFTSV were significantly smaller than those created by parental rVSV (Figure 1E). Taken together, these results suggest that rVSV-SFTSV is attenuated in cell culture and replicates with slower kinetics than parental rVSV.

**Figure 2:**
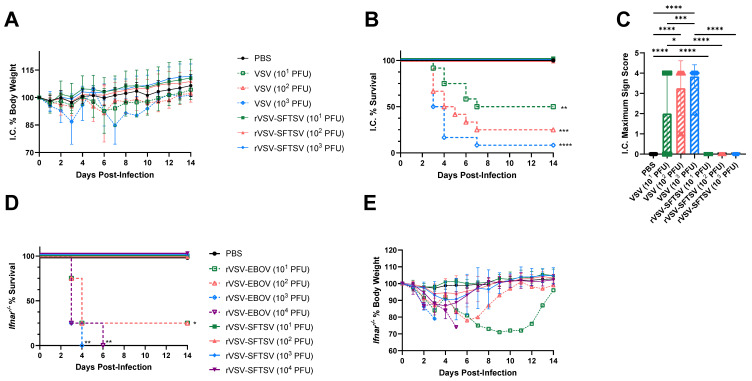
rVSV-SFTSV has a favorable safety profile compared to rVSV-EBOV and parental VSV. (**A**) Weight change, (**B**) survival proportions, (**C**) and maximal neurologic disease severity score in C57BL/6 mice challenged intracranially (IC) with 10^1^, 10^2^, or 10^3^ PFU of parental VSV or rVSV-SFTSV into the right cerebral hemisphere (Mantel–Cox test and ordinary one-way ANOVA; *, *p* < 0.0332; **, *p* < 0.0021; ***, *p* < 0.0002; ****, *p* < 0.0001). (**D**) Survival proportions and (**E**) weight loss of *Ifnar^−/−^* mice challenged intraperitoneally with PBS or 10^1^, 10^2^, 10^3^, or 10^4^ PFU of either rVSV-SFTSV or rVSV-EBOV. Weight changes were reported as percentages of body weight measured immediately pre-challenge. (Mantel–Cox test; *, *p* < 0.0332; **, *p* < 0.0021).

One safety concern with rVSV vaccines is the potential for neurotropism [91,92]. VSV-G, the cognate glycoprotein of VSV, is sufficient to catalyze viral entry into neurons, and VSV can replicate efficiently in neuron-like cells both in vitro and in vivo [78,93]. In addition, other rhabdoviruses, such as rabies virus, are known human pathogens that cause lethal neurotropic infections [94]. It is currently unclear whether neurons can be infected by SFTSV or viruses harboring SFTSV glycoproteins. Neurologic symptoms have been observed in human SFTS cases, but it remains unclear whether the SFTSV glycoproteins can initiate entry into neurons in vivo [95]. To test the neuropathogenic potential of rVSV-SFTSV, we injected escalating doses of rVSV-SFTSV or VSV Indiana strain intracranially into the right cerebral hemisphere of 4-week-old C57BL/6 mice and observed the mice for 14 days. All groups of mice lost 2–5% body weight the day following intracranial injection independent of inoculum composition (Figure 2A). Weight loss in mice injected with rVSV-SFTSV was reversed by 4 days post-infection (dpi). In contrast, weight loss was more severe in mice challenged with VSV and survivors exhibited slower recovery. None of the mice injected with rVSV-SFTSV met humane endpoints during the experiment (Figure 2B). By comparison, lethal disease was seen in mice injected with VSV that trended with an inoculum dose. Neurologic effects were quantified by using a neurologic sign scoring scale that ranged from 0 (no neurologic signs) to 4 (severe neurologic signs). No neurologic signs were observed in mice inoculated with rVSV-SFTSV or vehicle. In contrast, a range of neurologic manifestations were observed in most mice injected with VSV (Figure 2C). All the observed neurologic effects occurred between 2–7 dpi.

The rVSV-EBOV vaccine received FDA approval despite severe pathogenicity in *Ifnar^−/−^* and *Stat1^−/−^* immunocompromised mice [91,92]. To evaluate the pathogenicity of rVSV-SFTSV, we challenged groups of *Ifnar^−/−^* mice with escalating doses of rVSV-SFTSV or rVSV-EBOV. All mice injected with rVSV-SFTSV were alive 14 dpi (Figure 2D). In contrast, at least 50% of mice infected with rVSV-EBOV met humane endpoints by 4 dpi, and all mice challenged with at least 10^3^ PFU succumbed by 6 dpi. All groups of mice challenged with rVSV-EBOV exhibited weight loss beginning 2 dpi, which progressed with time (Figure 2E). The surviving mice challenged with rVSV-EBOV lost at least 20% body weight prior to their recovery. Mice challenged with rVSV-SFTSV also began losing weight by 2 dpi, but the weight loss was less severe compared to rVSV-EBOV groups. Of the groups of mice challenged with rVSV-SFTSV, only the group infected with 10^4^ PFU lost more than 10% body weight. Recovery from weight loss began between 4–5 dpi for all mice challenged with rVSV-SFTSV. Collectively, these experiments showed that rVSV-SFTSV was not neuropathogenic and demonstrated a significantly more favorable safety profile in immunocompromised mice than the currently approved rVSV-EBOV vaccine.

### 3.2. Single Vaccination with rVSV-SFTSV Induces High Levels of Neutralizing Antibodies

**Figure 3:**
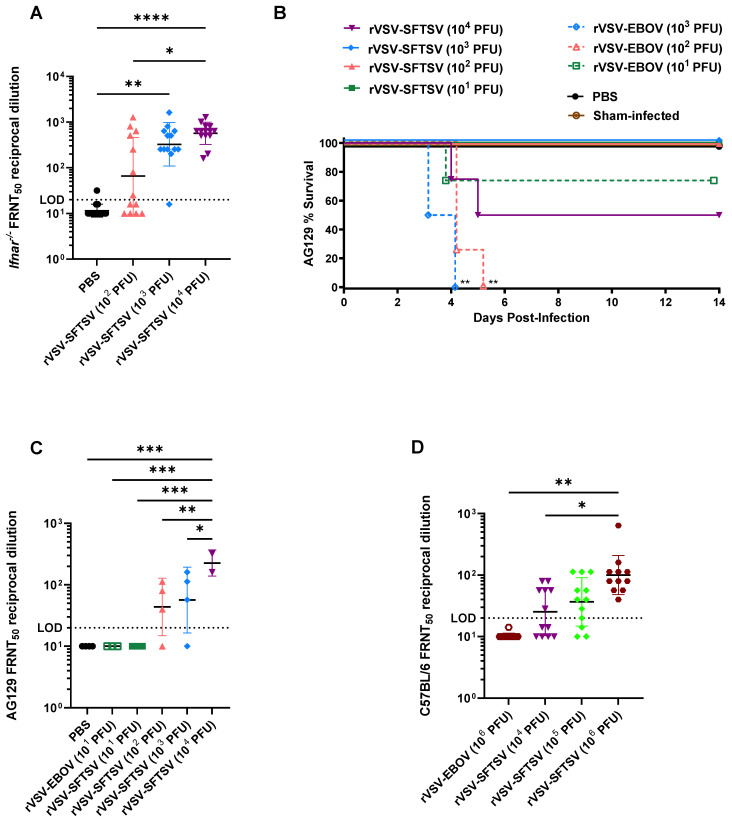
rVSV-SFTSV induces neutralizing antibodies across different mouse strains. (**A**) *Ifnar^−/−^* mice were immunized with PBS, 10^2^, 10^3^, or 10^4^ PFU rVSV-SFTSV. Serum neutralizing antibodies were quantified by measuring 50% decrease in pseudovirus foci, the reciprocal endpoint dilution is shown (Ordinary one-way ANOVA; *, *p* < 0.0332; **, *p* < 0.0021; ****, *p* < 0.0001). (**B**,**C**) AG129 mice were vaccinated with varying doses of rVSV-SFTSV and monitored for survival (**B**) and had serum collected 21 days post vaccination and FRNT_50_ was assessed (**C**) (Mantel–Cox test and ordinary one-way ANOVA; *, *p* < 0.0332; **, *p* < 0.0021; ***, *p* < 0.0002). (**D**) Wild-type C57BL/6 mice were immunized with rVSV-SFTSV and had serum neutralization titers determined at 21 days post treatment (Ordinary one-way ANOVA; *, *p* < 0.0332; **, *p* < 0.0021). Horizontal dotted lines indicate the limit of detection (LOD) of the assay.

To functionally characterize humoral responses to rVSV-SFTSV vaccination, we assessed antibody neutralization potential by focus reduction neutralization titer of 50% (FRNT_50_) in several animal models. To assess whether the *Ifnar^−/−^* mouse model could mount an antibody response, mice were immunized IP with 10^2^, 10^3^, or 10^4^ PFU of rVSV-SFTSV. At 21 dpi, sera were collected and analyzed for FRNT_50_. Approximately half of the animals vaccinated with 10^2^ PFU failed to seroconvert. Increasing the vaccination dose increased rates of seroconversion and neutralization titers (Figure 3A). These titers are promising given that previous work on influenza and SARS-CoV-2 suggest that neutralizing titers of 40–80 are sufficient for protection [96,97].

The high levels of neutralizing antibodies achieved with vaccination of *Ifnar^−/−^* mice was somewhat surprising as interferons (IFNs) are important drivers of immune responses. To determine whether mice deficient in both type I and type II IFN receptors also elicit high levels of neutralizing antibodies, we immunized AG129 mice with 10^1^–10^4^ PFU of rVSV-SFTSV. Notably, 2 of 4 mice immunized with 10^4^ PFU rVSV-SFTSV succumbed to viral infection (Figure 3B). Mice receiving 10 PFU rVSV-SFTSV failed to generate a neutralizing antibody response (Figure 3C). Animals receiving higher doses had mean neutralizing titers ranging from 60 to 240 with increasing dosage (Figure 3C). These results demonstrate that rVSV-SFTSV elicits humoral responses even in highly immunocompromised animals lacking type I and II IFN responses.

It is well documented that VSV infection is highly sensitive to IFN responses [98,99]. To determine whether rVSV-SFTSV can induce a neutralizing antibody response in mice with an intact interferon response, we immunized C57BL/6 mice with 10^4^, 10^5^, or 10^6^ PFU of rVSV-SFTSV. Dosages were increased relative to *Ifnar^−/−^* mice to account for IFN responses limiting rVSV-SFTSV replication and thus reducing the humoral immune response in the immune competent mice. In contrast to what was seen with the immune deficient mice, no weight loss was observed in the C57BL/6 mice at any vaccine dose. Additionally, despite the increased dosages, neutralizing titers were far lower than those observed in *Ifnar^−/−^* mice, suggesting that rVSV-SFTSV is sensitive to IFN, which is consistent with previous reports (Figure 3D). A dosage dependent increase in FRNT_50_ titers was observed with mice receiving 10^6^ PFU rVSV-SFTSV, achieving a mean titer of 113 (Figure 3D). Notably, all mice immunized with 10^6^ PFU rVSV-SFTSV seroconverted (Figure 3D). These data indicate that despite VSV’s sensitivity to IFN, rVSV-SFTSV induces responses in immunocompetent animals that reach neutralizing titers predicted to be protective.

### 3.3. rVSV-SFTSV Protects Ifnar^−/−^ Mice from Lethal SFTSV Challenge and Reduces Viral Titers in Tissues

**Figure 4:**
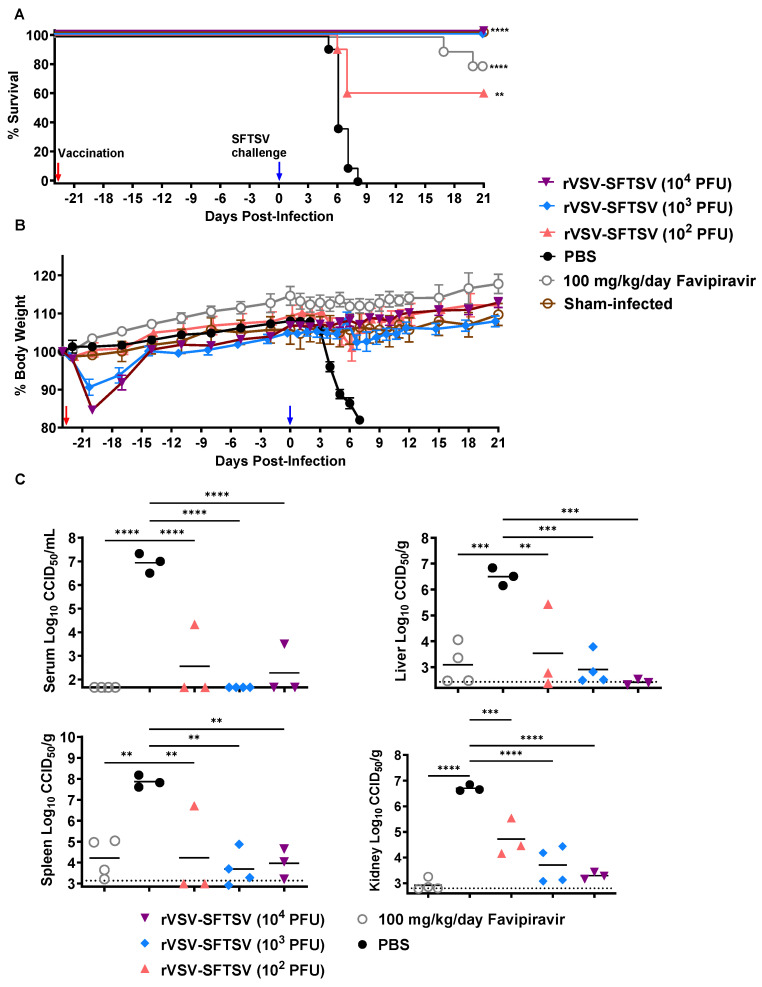
Vaccination with rVSV-SFTSV protects *Ifnar*^*−/−*^ mice from lethal SFTSV challenge. (**A**) Survival proportions and (**B**) percent weight change in *Ifnar^−/−^* mice challenged subcutaneously with 10 PFU SFTSV (blue arrow) 23 days after IP vaccination with PBS, or 10^2^, 10^3^, or 10^4^ PFU rVSV-SFTSV (red arrow). Weight change is reported as percentage change in body weight relative to starting weight prior to vaccination. One group of mice received favipiravir daily for eight days following SFTSV challenge to serve as a positive control for protection. (Mantel–Cox test; **, *p* < 0.0021; ****, *p* < 0.0001). (**C**) SFTSV titers in serum liver, spleen, and kidney five days post-challenge from mice subjected to the same vaccination schedule as those in (**A**,**B**). Horizontal dotted lines indicate the limit of detection of the assay (Ordinary one-way ANOVA; **, *p* < 0.0021; ***, *p* < 0.0002; ****, *p* < 0.0001).

Because of the high neutralizing antibody titers measured in *Ifnar^−/−^* mice vaccinated with rVSV-SFTSV, we hypothesized that the vaccine would protect these mice against lethal SFTSV challenge. To test this hypothesis, vaccinated mice were challenged subcutaneously with 10 PFU of SFTSV strain HB29 23 days post-vaccination (2 days after blood collection for neutralizing antibody titration). A single group of unvaccinated mice received 8 days of 100 mg/kg/day favipiravir therapy following SFTSV challenge as a positive control for protection [100]. As expected, all mice vaccinated with PBS succumbed by 8 dpi (Figure 4A). In contrast, 60% of mice vaccinated with 10^2^ PFU, and all *Ifnar^−/−^* mice vaccinated with at least 10^3^ PFU, survived the lethal SFTSV challenge. Mice vaccinated with at least 10^3^ PFU were protected from weight loss following SFTSV challenge while rapid weight loss was observed in PBS-vaccinated mice beginning by 3 dpi (Figure 4B). Mild weight loss occurred post challenge in mice that received 10^2^ PFU of vaccine, but this trend was driven primarily by the three individuals that succumbed to disease. Vaccination-associated weight loss was also observed in this experiment and was consistent in magnitude with that shown previously (Figure 2E).

To assess the effect rVSV-SFTSV vaccination has on SFTSV viremia and tissue viral loads, groups of 4 mice were vaccinated with escalating doses of rVSV-SFTSV and challenged in parallel 23 days post vaccination, according to the timeline described above. These subsets of mice were sacrificed 5 days following SFTSV challenge and serum, liver, spleen, and kidney were collected for SFTSV quantification by endpoint titration on Vero E6 cells. All groups of vaccinated mice had significantly reduced SFTSV serum and tissue viral titers (Figure 4C). In the liver and kidney, there was a trend towards dose-dependence with mice vaccinated with the highest dose of rVSV-SFTSV having the lowest viral titers. Favipiravir treatment also reduced SFTSV titers compared to mice vaccinated with PBS. These data demonstrate that rVSV-SFTSV does not provide sterilizing immunity to SFTSV challenge but rather reduces replication in the vaccinated animals at day five post challenge.

### 3.4. Passive Serum Transfer Imparts Protective Immunity to Naïve Mice

**Figure 5:**
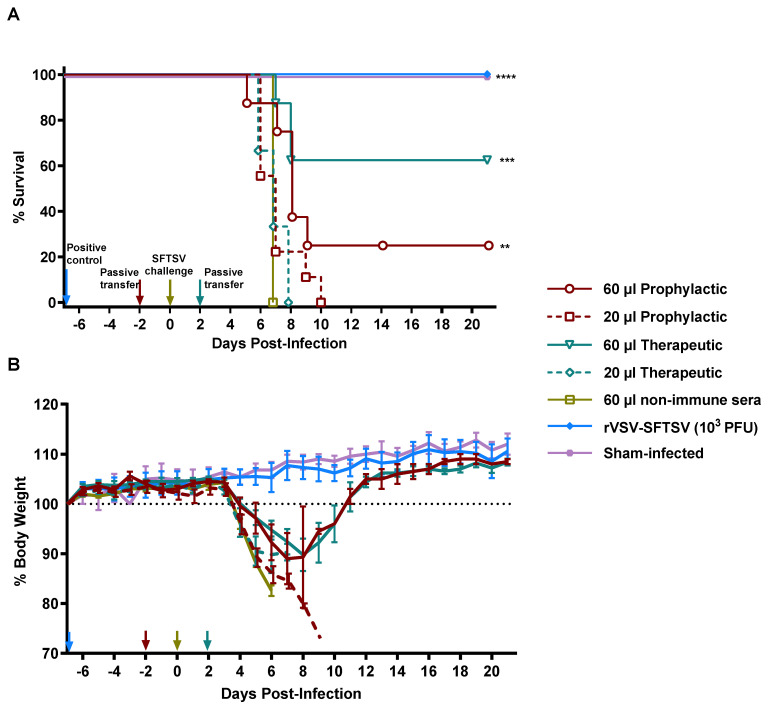
Passive transfer of sera from immunized mice protects naïve mice against SFTSV challenge. Survival (**A**) and weight loss (**B**) curves are shown from naïve animals receiving immune sera either 2 days prior to or 2 days post challenge with 10 PFU of SFTSV. Mice immunized with 10^3^ PFU of the rVSV-SFTSV 7 days prior to challenge served as the positive control. Blue arrow, immunization with rVSV-SFTSV 7 days prior to challenge; Red arrow, passive transfer 2 days prior to challenge; Yellow arrow, SFTSV challenge; Teal arrow, passive transfer 2 days post SFTSV challenge. (Mantel–Cox test; **, *p* < 0.0021; ***, *p* < 0.0002; ****, *p* < 0.0001).

Our data demonstrate that rVSV-SFTSV vaccination induces a neutralizing antibody response and protects against lethal SFTSV infection. Previous studies of SFTSV monoclonal antibodies and nanobodies have demonstrated that they can provide protection in mouse models [32,33]. To assess whether antibodies induced by rVSV-SFTSV vaccination impart protection against lethal SFTSV infection, we performed passive serum transfer. To begin, 60 µL or 20 µL of sera from rVSV-SFTSV-immunized or negative control *Ifnar^−/−^* mice were administered to naïve *Ifnar^−/−^* mice either prophylactically (2 days prior to challenge) or therapeutically (2 days post challenge). The pooled sera used for passive transfer had an approximate FRNT_50_ titer of 450, while the FRNT_50_ titer for negative control sera was below the limit of detection. Approximately 33% of *Ifnar^−/−^* mice receiving 60 µL of immune sera prophylactically were protected against lethal SFTSV challenge (Figure 5A). When 60 µL of immune sera was administered therapeutically, 62% of mice survived (Figure 5A). Animals given 20 µL of immune sera were not protected from challenge regardless of when sera were administered (Figure 5A). It is likely that the animals that succumbed to challenge did not receive sufficient antibodies for protection. All mice in the positive control group receiving 10^3^ PFU rVSV-SFTSV 7 days prior to challenge survived (Figure 5A).

Animal weights measured daily during the study positively correlated with the survival data (Figure 5B). The most dramatic weight loss after the SFTSV challenge occurred in the group treated with the non-immune sera and the groups treated with the lower quantities of immune sera. All surviving mice treated with the 60 µL dose of immune sera recovered fully from the infection after losing approximately 10% body weight (Figure 5B). The mice vaccinated with 10^3^ PFU rVSV-SFTSV did not experience any weight loss due to the vaccine virus or upon SFTSV infection (Figure 5B).

### 3.5. rVSV-SFTSV Vaccination Cross-Protects Against Lethal HRTV Challenge

**Figure 6:**
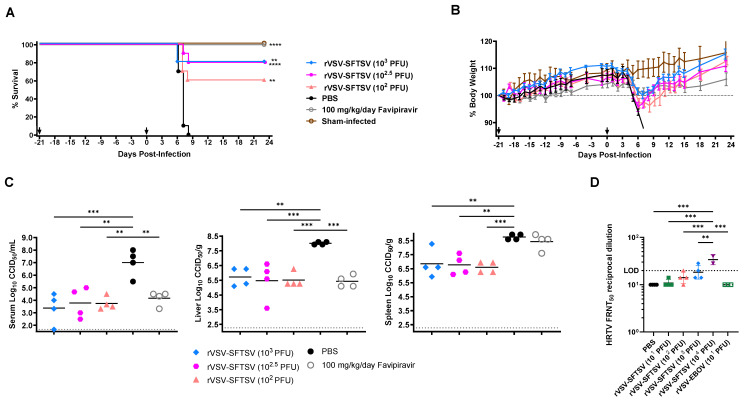
rVSV-SFTSV vaccination cross-protects animals against MA-HRTV challenge. AG129 mice were IP immunized with escalating doses of rVSV-SFTSV then challenged with MA-HRTV 21 days post immunization. (**A**) Survival and (**B**) weight loss curves are shown from immunization until completion of experiment. Black arrows indicate vaccination and challenge times at −21 and 0 days respectively (Mantel–Cox test; **, *p* < 0.0021; ****, *p* < 0.0001). (**C**) Four animals in each vaccination group were sacrificed 5 days post challenge to assess serum, liver, and spleen virus titers (Ordinary one-way ANOVA; **, *p* < 0.0021; ***, *p* < 0.0002) (**D**) Sera was collected from subsets of animals 21 days post immunization and prior to HRTV challenge. Sera was analyzed for neutralizing antibodies against HRTV using a pseudotyped virus with the HRTV Gn/Gc glycoprotein. Horizontal dotted lines indicate the limit of detection (LOD) of the assay (Ordinary one-way ANOVA; **, *p* < 0.0021; ***, *p* < 0.0002).

Next, we evaluated whether vaccination with rVSV-SFTSV confers cross protection against challenge with the related HRTV. In IFN-α/β and γ receptor-deficient AG129 mice, a dose of 10^4^ PFU rVSV-SFTSV was partially lethal (Figure 3B). Thus, we modified immunization dosages to 10^2^, 10^2.5^, and 10^3^ PFU rVSV-SFTSV. Mice were then challenged with a lethal dose of a mouse-adapted HRTV (MA-HRTV) 21 days post vaccination [87]. A group of unvaccinated mice were treated with 100 mg/kg/day favipiravir for 8 days following MA-HRTV challenge. Eighty percent of the mice that received the two highest doses of 10^2.5^ or 10^3^ PFU rVSV-SFTSV survived the challenge, with 60% of the mice vaccinated with the lowest dose (10^2^ PFU) also surviving (Figure 6A). As expected, all PBS-vaccinated mice succumbed to MA-HRTV disease by 8 dpi and all the favipiravir-treated animals were protected (Figure 6A). Most of the infected mice experienced considerable weight loss beginning 4 to 6 days post MA-HRTV challenge (Figure 6B). Surviving mice fully recovered and had weight gain comparable to favipiravir-treated animals.

Subsets of 4 mice per experimental group were sacrificed on day 5 post MA-HRTV challenge for collection of blood, liver, and spleen tissue for measurement of viral loads by endpoint titration using an infectious cell culture assay. Mice immunized with rVSV-SFTSV had significantly reduced MA-HRTV titers comparable to the favipiravir positive control (Figure 6C). Serum collected from vaccinated animals in the initial immunogenicity study was analyzed for neutralizing activity. Mice vaccinated with 10^2^ or 10^3^ PFU rVSV-SFTSV had moderate SFTSV neutralization titers of 40 and 60, respectively, with one mouse in each group failing to seroconvert (Figure 3C). Mice receiving 10^4^ PFU immunization doses reached higher neutralization titers against SFTSV but 2 of 4 animals succumbed to the vaccine virus (Figure 3B,C). In contrast, cross-neutralization of HRTV was only observed in sera from 2 of 4 mice receiving 10^3^ PFU immunizations. HRTV neutralizing activity was also seen in sera from the surviving 10^4^ PFU immunized animals from the safety and immunogenicity study (Figure 6D). In mice immunized with 10^3^ PFU rVSV-SFTSV, neutralizing titers against HRTV were at or just above the limit of detection indicating weak cross reactivity (Figure 6D). Sera from mice receiving lower immunization doses did not have neutralization activity despite partial protection from MA-HRTV challenge (Figure 6A,C,D). Lack of cross neutralization titers suggests that the protective effect in the context of survival and reduced viral loads may be due to cell-mediated immunity or other non-neutralizing antibody functions.

## 4. Discussion

SFTSV and HRTV are emerging tick-borne viruses with high fatality rates, and their significance to public health is emphasized by prioritization by the WHO and NIAID [56,57]. Despite their designation as priority pathogens, no vaccines or therapeutics have been approved for either virus by the FDA. Much work has been done to address the lack of available SFTSV vaccines, but the majority of the developed candidates have potential drawbacks [101]. DNA vaccines encoding the SFTSV glycoprotein were among the earliest vaccines to be developed and tested in animal models, and they were protective against lethal challenge in ferrets [58] and IFNAR knockout mice when an IL-12 adjuvant was included in the vaccine [59]. However, DNA vaccination required in vivo electroporation in animal models, limiting the broad translation to human clinical studies, especially in rural areas where tick exposure is most common but public health infrastructure is less reliable [102,103]. Whole inactivated SFTSV virus vaccines have also been trialed in C57BL/6 mice, but three doses were required for protection against SFTSV challenge [65]. Live-attenuated viruses have also been developed as a vaccine candidate, and vaccines were safe and effective in immunized ferrets [66]. However, concerns still exist about the possibility of reversion to virulence in elderly or immunocompromised people, the group most susceptible to SFTSV infection [103]. Other vaccine candidates that have been explored in animal models include protein subunit vaccines using the SFTSV non-structural protein [63] or glycoproteins [59], but neither showed protective efficacy. The only protein subunit vaccine to be protective in animal models requires the Gn glycoprotein to be fused to a ferritin nanoparticle, which carries a high cost of production [64].

The two vaccine platforms that show the greatest promise for SFTSV are mRNA and rVSV-SFTSV [60,61,62,67,104]. Studies have shown that mRNA vaccines encoding SFTSV Gn, Gn head, or Gn head fused to a self-assembling ferritin nanoparticle induced efficient humoral and cellular immunity, and vaccination was protective against lethal SFTSV challenge [60,61]. An additional study showed that mRNA encoding the full-length SFTSV glycoprotein was also highly effective against SFTSV and conferred additional crossprotection against HRTV and another closely related Guertu virus [62,105]. A significant advantage to mRNA vaccines is their favorable safety profile as safety is a concern for all live attenuated viruses or viral-vectored vaccines. However, our data show that rVSV-SFTSV is safe even in severely immunocompromised mice and does not result in significant morbidity. Additionally, the SFTSV mRNA vaccines tested thus far require two doses for complete protection, and evidence collected from humans vaccinated against SARS-CoV-2 suggests three or more doses may be required to induce robust memory responses [60,61,62,106]. Additionally, distribution and storage of mRNA vaccines may be limited in rural communities lacking robust public health systems due to the dependence of mRNA vaccines on extremely low temperature storage to remain viable [107,108]. rVSV-SFTSV vaccines have previously been shown to induce humoral and cellular immunity and be highly protective for SFTSV [67]. Additionally, data from studies on the rVSV vaccine for Ebola virus (rVSV-EBOV) in immunized humans and animal models demonstrated a potentially unique characteristic of rVSV vaccines in that they may be protective when administered post-exposure under certain circumstances [109,110]. The tickborne nature of SFTSV, HRTV, and related bandavirus infections makes these viruses highly suitable for post-exposure prophylaxis. To date, there is no evidence that mRNA vaccines are protective post-exposure [108]. The effective use of the commercial rVSV-EBOV vaccine Ervebo in inaccessible, under-resourced areas in Africa [84] also suggests that a rVSV-SFTSV vaccine could be of utility in the rural areas of Southeast Asia where SFTSV is endemic [103]. Historically, VSV vaccines have relied on extreme cold chain storage and transportation, similar to mRNA vaccines. However, advances in storage technology for VSV vaccines have greatly increased the stability of the vaccine, limiting the requirements for cold chain infrastructure [111]. Importantly for vaccines used in inaccessible or under-resourced areas, our results demonstrate that a single dose of rVSV-SFTSV is completely protective against SFTSV lethal challenge. To date, there has not yet been a study that directly compares the immune responses elicited by an mRNA encoding SFTSV glycoproteins against those raised by rVSV-SFTSV. Nonetheless, the characteristics of rVSV vaccines provide compelling clinical and economic incentives for further development of rVSV-SFTSV and related vaccines.

The most prominent concern of viral-vectored vaccines is safety, especially in elderly and immunocompromised populations. Indeed, the only currently FDA-approved rVSV vaccine, rVSV-EBOV, is highly pathogenic and lethal in *Ifnar^−/−^* mice. In contrast, our rVSV-SFTSV vaccine only caused mild-to-moderate weight loss at doses that elicited protective immunity. Importantly, unlike the parental VSV vector, rVSV-SFTSV did not cause neurologic disease when injected intracranially into 4-week-old C57BL/6 mice, suggesting that this vaccine strain is not neurotropic. These data suggest that rVSV-SFTSV has a low potential for pathogenicity and a positive safety profile, even in immunocompromised populations. However, despite these promising results, it is possible that rVSV-SFTSV may be too attenuated in animals containing a functional IFN system. This possibility is supported by the lower neutralizing antibody titers measured in vaccinated C57BL/6 mice compared to those in *Ifnar^−/−^* mice. Critically, our passive transfer experiment demonstrated protection in *Ifnar^−/−^* mice in which the calculated neutralizing antibody titer would be below the limit of detection of our neutralization assay. Additionally, neutralizing serum levels in vaccinated C57BL/6 mice were higher than titers that are typically expected to protect against other viruses such as influenza, which suggests that rVSV-SFTSV may be protective in immunocompetent hosts [96].

While the safety profile of rVSV-SFTSV is extremely positive and suggests that it can be safely used in immunocompromised hosts, one potential drawback of rVSV-SFTSV is that it is highly attenuated in cell culture, as shown by our data and previous work [67]. This attenuation likely results from the increased size of the SFSTV glycoprotein relative to the native VSV-G glycoprotein in addition to the natural tendency of SFTSV and other bunyaviruses to bud from the Golgi apparatus and ERGIC [75,112,113]. Viral attenuation may limit immunogenicity by impairing replication in animal models, and it makes it difficult to produce sufficient amounts of the virus to initiate large scale clinical trials. One previous study has identified two mutations (C617R and M749T) in the SFTSV glycoprotein that occur spontaneously during rVSV-SFTSV replication and significantly increase the viral titer [104]. In this study, we have identified two additional mutations, E982K and K1071E, that may similarly increase viral replication. Nevertheless, it is important to balance the replication efficiency and resulting immunogenicity of the virus with the safety of the vaccine candidate. Future work will analyze the specific mechanisms of these mutations, the potential for combination into one vaccine candidate, and the safety profile of the resulting vaccine.

Neutralizing antibodies elicited in the context of a functional humoral response are known to be a correlate of protection against lethal SFTS in humans [71]. As such, passive transfer of sera from vaccinated animals has been previously shown to be protective when used prophylactically in an *Ifnar^−/−^* mouse model of lethal SFTSV [67]. Extending these findings, our results demonstrated that serum from mice vaccinated with rVSV-SFTSV could protect against lethal SFTSV challenge when used either prophylactically or therapeutically, suggesting the protective capacity of Gn/Gc-targeted antibodies against SFTSV. In support of the role for antibodies in protection against SFTS disease, monoclonal antibodies and nanobodies recognizing Gn protect *Ifnar^−/−^* and NCG-HuPBL mice from lethal challenge and thrombocytopenia, respectively [32,33]. Furthermore, development of bispecific antibodies targeting both Gn and Gc has improved the potency, resulting in increased protection at lower doses [114]. Our study contributes to knowledge of the efficacy of neutralizing sera administered therapeutically, which has implications for monoclonal antibody or antibody cocktail management of patients with SFTS. Protective functions of antibodies against SFTSV outside of neutralization were beyond the scope of our studies and warrant future investigation.

In addition to direct neutralization by elicited antibodies, our data also suggest other mechanisms for protection against SFTSV pathogenesis. In our passive transfer studies, following redistribution of transferred sera from the peritoneal cavity to the circulation and subsequent dilution within the host’s blood, it is likely that the neutralizing titer of the recipient’s sera would be below the limit of detection of our FRNT_50_ assay. Still, the dose of antibodies contained within 60 μL of donor sera was sufficient to protect mice under both prophylactic and therapeutic conditions. Surprisingly, we observed better protection when sera were transferred therapeutically (2 dpi) compared to prophylactically (−2 dpi). While the mechanisms underlying this observation are unknown, it is possible that uncharacterized variables such as the pharmacokinetics and bioavailability of the sera components responsible for protection at sites of virus replication may be responsible. In addition, our results suggest that passive transfer intervention may be beneficial in areas endemic for SFTSV within high-risk populations for severe SFTS that have reported recent tick bites but are yet to show symptoms. Further studies using appropriate animal models should be conducted to better evaluate the temporal relationship of passive transfer therapies with both infection (by experimental inoculation or tick bite) and clinical disease onset.

Protection against lethal challenge in the absence of high neutralizing antibody titers has been observed in SFTSV vaccines using recombinant vaccinia virus and DNA technologies [59,68]. These observations suggest that other mechanisms in addition to neutralizing antibodies may also contribute to protection against SFTS. In this study, we demonstrated that vaccination with rVSV-SFTSV either 7 or 21 days prior to SFTSV challenge was fully protective (Figure 4A and Figure 5A). Protection at 7 days, a time at which humoral responses are not fully developed, suggests that mechanisms other than antibodies can be protective. Since the T cell immune response peaks at approximately 7 days post-vaccination, it is possible that these cells also contribute to the protection elicited by the vaccine [115,116]. Additionally, we noted protection against SFTSV lethality in some of the rVSV immunized mice where the neutralizing titer was below the limit of detection. rVSVs are known to stimulate robust T_H_1 immune responses in vaccinated animals and humans [117,118,119]. The data presented here suggest that rVSV-SFTSV may also confer protection by inducing type 1 immunity and CD8+ T cells. It is also possible that NK cells or other components of the innate immune system are important mediators of this early protective effect of the rVSV vaccine. More studies will be required to elucidate the potential role of T cells following vaccination with rVSV-SFTSV or infection by authentic SFTSV.

Previous studies have shown that sera raised by vaccination against SFTSV glycoproteins can cross-neutralize viruses harboring HRTV glycoproteins [67]. In addition, this same study showed that a rVSV-HRTV protects *Ifnar^−/−^* mice against lethal SFTSV infection. The present study is the first to report that rVSV-SFTSV protects *Ifnar^−/−^* mice against a lethal HRTV challenge. Despite protection against lethal disease, sera from rVSV-SFTSV-vaccinated mice neutralized viruses pseudotyped with HRTV glycoproteins much less efficiently than viruses pseudotyped with SFTSV glycoproteins. Of the four serum samples collected from AG129 mice vaccinated with 10^2^ PFU of rVSV-SFTSV, only one had a detectable neutralizing antibody titer (reciprocal dilution of 20) against VSV pseudotyped with HRTV glycoproteins. Despite this, 60% of AG129 mice that received this dose of vaccine survived MA-HRTV challenge. These data suggest that other systems stimulated by rVSV-SFTSV vaccination, such as a T_H_1 response, could have contributed to the protection against HRTV. Additionally, alternative effector functions of antibodies beyond neutralization may contribute to the protective effect described here. Further studies are needed to elucidate which immune system components are responsible for cross-protection as the results of these studies could inform future vaccine development for these recently emerged bandaviruses.

## Data Availability

All datasets generated for this study are included in the article. Raw data will be furnished upon request.

## Supplementary Materials

The following supporting information can be downloaded at: https://www.mdpi.com/article/10.3390/vaccines12121403/s1, The uncropped blots are shown in Supplementary Materials.
