# Supplementary figures and images for: Safety, Immunogenicity, and Efficacy of a Recombinant Vesicular Stomatitis Virus Vectored Vaccine Against Severe Fever with Thrombocytopenia Syndrome Virus and Heartland Bandavirus
Source: Vaccines (Basel). 2024 Dec 12;12(12):1403. doi: 10.3390/vaccines12121403 (PMC11728676; doi:10.3390/vaccines12121403)

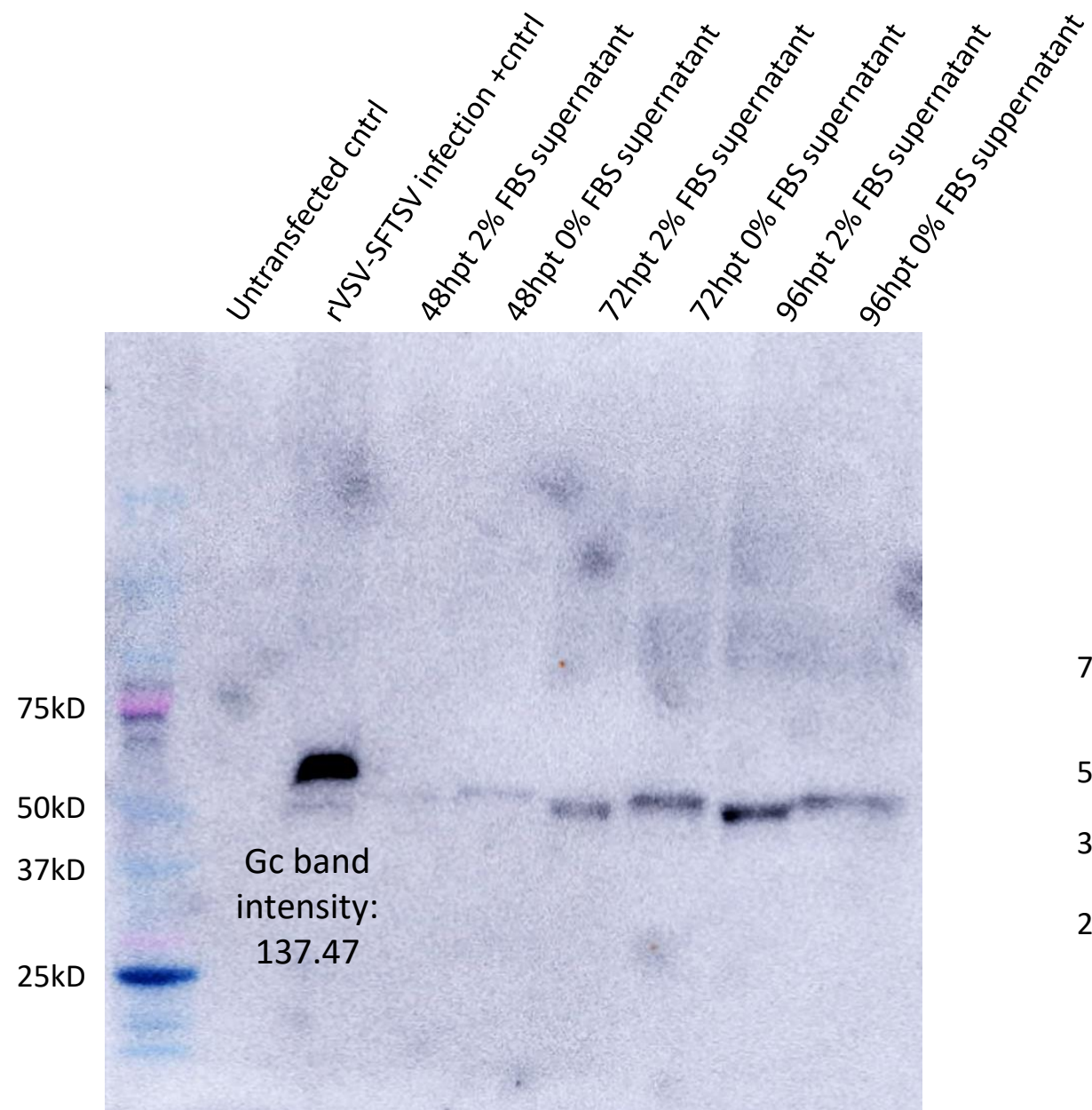

Gc western blot

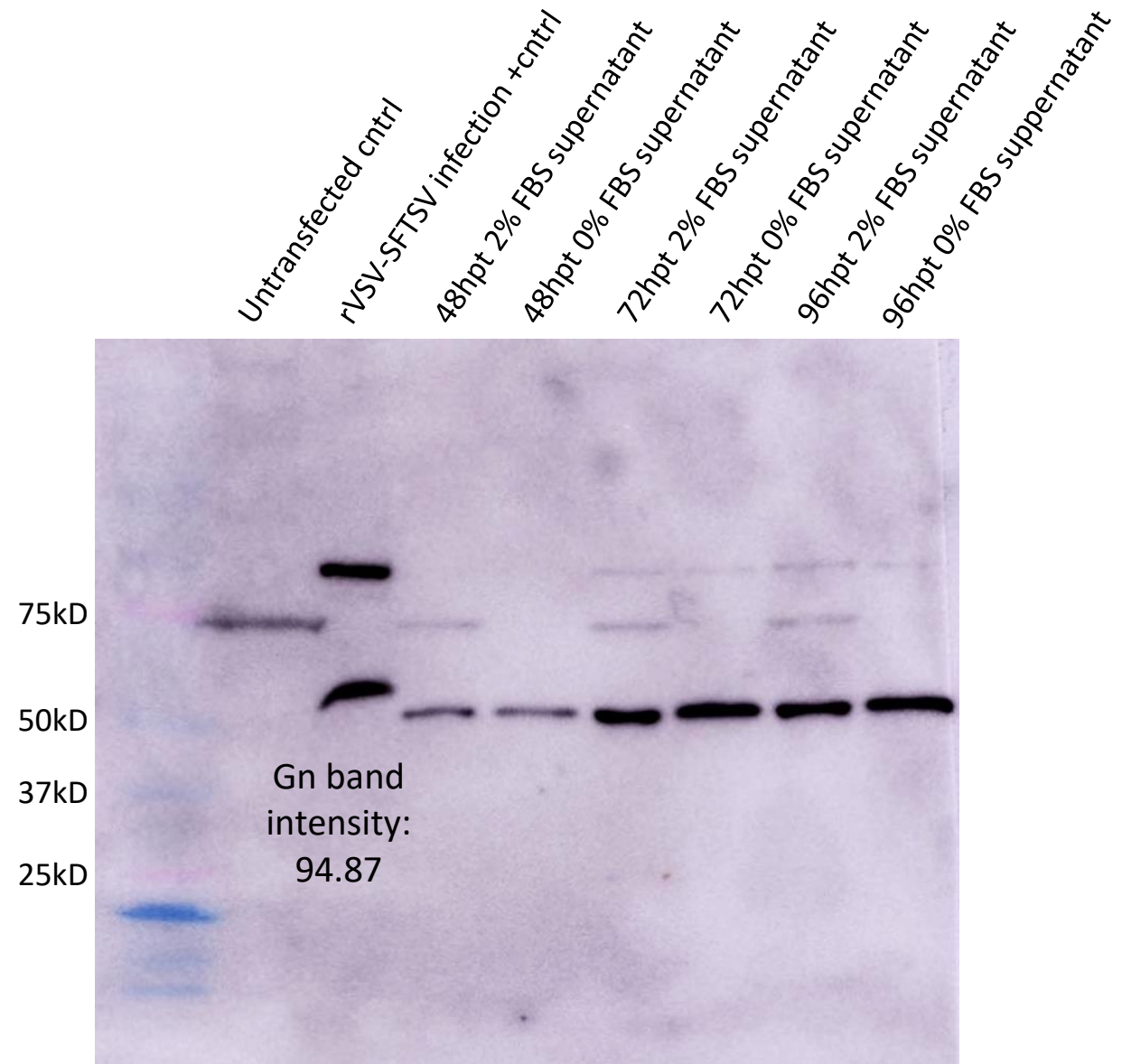

Gn western blot

Supplement: Supplementary file 1 [file vaccines-12-01403-s001.zip › vaccines-3355285-supplementary.pdf]
